# Accurate Visualization of C4d Complement Fragment in Immunohistochemistry by C-Terminal Linear Neoepitope-Specific Antibodies

**DOI:** 10.3390/ijms251910526

**Published:** 2024-09-30

**Authors:** Daria Kowalska, Michał Bieńkowski, Paulina Jurkowska, Ada Kawecka, Jacek Kuryło, Alicja Kuźniewska, Marcin Okrój

**Affiliations:** 1 Department of Cell Biology and Immunology, Intercollegiate Faculty of Biotechnology, University of Gdańsk and Medical University of Gdańsk, Dębinki 1 Street, 80-211 Gdańsk, Poland; daria.kowalska@gumed.edu.pl (D.K.); p.jurkowsk1a@gmail.com (P.J.); ada.kawecka@gumed.edu.pl (A.K.); jacek.kurylo99@gmail.com (J.K.); alicja.kuzniewska@gumed.edu.pl (A.K.); 2 Department of Pathomorphology, Medical University of Gdańsk, Smoluchowskiego 17 Street, 80-214 Gdańsk, Poland; michal.bienkowski@gumed.edu.pl

**Keywords:** C4d staining, immunohistochemistry, antibody-mediated rejection, complement C4d

## Abstract

C4d is the end degradation product of activated complement component C4b that appears during the early steps of the classical and lectin complement pathways. Within the primary sequence of C4d, there is a reactive thioester group that binds covalently to nearby surfaces, thus labeling the locations of complement activation. This feature makes C4d a target for immunohistochemical staining aimed to aid the diagnosis of, among others, the antibody-mediated rejection of transplanted organs, membranous glomerulonephritis, bullous pemphigoid, or inflammatory myopathies. However, the credibility of C4d immunostaining is debatable, as a high background in surrounding tissues and body fluids and diffused patterns of deposits in target structures are experienced with some of the available anti-C4d antibodies. Herein, we present an improved version of a rabbit anti-C4d antibody, originally raised against the C-terminal linear neoepitope of this complement fragment. Minor cross-reactivity with C4b and native C4 proteins, measured by ELISAs, as well as relatively low concentrations necessary for obtaining a specific signal in immunohistochemical analyses of formalin-fixed paraffin-embedded material, makes the improved antibody superior to commercially available rabbit monoclonal anti-C4d antibody SP91 dedicated to ex vivo diagnostics, as demonstrated by the staining of a panel of kidney transplant biopsies.

## 1. Introduction

Complement activation is a multi-step process that takes place either spontaneously by the breakdown of the C3 protein (alternative complement pathway, AP) or by specific stimuli such as antibodies (classical complement pathway, CP) and sugar moieties typical for microbes (lectin complement pathway, LP) sensed by pattern recognition molecules of the complement system (C1q or MBL/ficolins, respectively) [1]. CP is typically activated by human IgG and IgM antibodies that may appear as a humoral reaction to allografts. The sequence of classical pathway activation embraces antibody sensing by the C1q protein followed by the rearrangement of the C1 complex with the release of the enzymatic activity of C1r and C1s molecules. Activated C1s proenzyme cleaves serum C2 and C4 components into C2a/C2b and C4a/C4b, respectively [2]. On the one hand, C2a and C4b form classical C3 convertase, a labile enzymatic complex that mediates C3 breakdown and initiates the terminal stages of the complement cascade. On the other hand, convertases are tightly regulated by several complement inhibitors that boost their dissociation or proteolytic degradation [3]. The latter scenario involves a C4b fragment, which is converted by factor I (FI) and inhibitors with cofactor activity to both the iC4b form and eventually the C4d fragment [1]. Importantly, the amino acid sequence within the C4d fragment embraces an active thioester site, which is exposed upon the C4-C4b transition and binds C4b covalently to nearby surfaces [4]. Thereby, after complete C4b degradation, the end product C4d remains covalently attached to the cells that provoked complement activation. CP is activated mainly by human IgM and IgG antibodies that dominate primary and secondary humoral immune responses, respectively. This is why C4d was found useful as a molecular footprint of antibody-mediated graft rejection. C4d deposition is typically applied to the evaluation of kidney graft fate [5,6,7,8], but it has been also examined in cardiac [9,10,11], pancreas [12], lung [13], and face tissue transplants [14], as well as in the diagnostics of bullous pemphigoid [15], inflammatory myopathies [16], and glomerular diseases [17]. Technically, obtaining a specific anti-C4d antibody is not a trivial task due to its cross-reactivity with the native C4 protein and C4b fragment, which can be a common issue when immunizing with random C4d peptides or the whole C4d fragment. Previously, we described the process of raising rabbit polyclonal anti-C4d antibodies reactive to a five-amino-acid-long linear cleavage neoepitope located at the C-terminal part of the C4d fragment. To exclude the antibodies bondable to the region that spans both sides of the cleavage site, we first absorbed antibodies on Sepharose-immobilized peptides covering the immunogen (positive selection), and then such antibodies were cross-absorbed on peptides representing the intact cleavage site (negative selection). Our product showed very high specificity towards C4d in the ELISA, and unlike other commercially available anti-C4d antibodies, it did not generate a false positive signal when applied for the quantification of C4d in heat-inactivated serum or freeze–thawed serum samples [18]. Herein, we used a similar strategy for mapping the longest possible linear neoepitope that supports the formation of C4d-specific antibodies dedicated to the immunohistochemical analysis of human tissue. Such an antibody may become a more precise molecular tool than those already approved for ex vivo diagnostics. To prove our hypothesis, we compared its performance with the SP91 rabbit monoclonal antibody that is widely used by pathologists [19,20].

## 2. Results

First, we evaluated the specificity of rabbit polyclonal anti-C4d antibodies in two experimental setups: (i) direct ELISA, in which a purified C4d fragment (target antigen) and C4 and C4b proteins (as controls for cross-reactivity) were immobilized onto a plate (Figure S1A–E), and (ii) a setup in which a natural deposition of the classical complement pathway components from normal human serum took place (Figure S1F). When comparing the performance of rabbit antisera with purified antibodies, we can observe that the two-step purification process helped with reducing antibody cross-reactivity with C4 and C4b (Figure S1A–E), as antisera from certain rabbits showed significantly higher levels of C4b/C4 binding than purified antibodies of the same rabbit. Antibodies produced in the rabbits immunized with the constructs covering 5aa, 7aa, 9aa, 11aa, and 13aa epitopes that showed superior binding in direct ELISA were chosen for a further functionality test, i.e., detection of naturally deposited C4d. As expected, the antibodies were also capable of detecting C4d deposited on a plate after classical pathway activation, as visualized by the difference in signal from the wells coated with human antibodies and incubated with 1% normal human serum (NHS) and the same wells, to which FI-blocking antibody was added to impede C4d formation (Figure S1F). Heat-inactivated NHS and wells incubated with GVB++ buffer were considered as negative controls showing the background binding level. We concluded that the antibodies raised against the 11aa epitope showed cross-reactivity to other naturally deposited C4 forms, as evidenced by the substantial signal despite FI blocking. We also noticed a much weaker signal from the antibodies raised against the 13aa epitope compared to other antibodies. Since we aimed to select maximally long epitopes that ensure strong but specific binding to C4d, we chose the 7aa and 9aa epitopes as immunogens for the custom production of monoclonal antibodies.

After the initial screening tests, 3 out of 40 clones of monoclonal antibodies provided by Applied StemCell were selected: XS41, XS43, and SX4. A direct ELISA analogous to that performed for polyclonal antibodies showed no significant differences in binding to immobilized purified proteins/protein fragments. However, XS43 exhibited the lowest cross-reactivity (Figure S2A) and lowest working concentration. To extend the setup of the other ELISA checking the binding specificity to C4d deposited from serum, next to the application of FI-blocking antibodies, we added two conditions: FI-depleted serum (ΔFI), where the lack of FI disables C4b to C4d processing, and ΔFI + FI, where serum is reconstituted with purified FI, creating conditions similar to NHS. Surprisingly, the XS41 clone failed to bind to its target antigen in these conditions, whereas the SX4 clone showed a high signal in ΔFI serum (Figure S2B). Therefore, the best-performing clone XS43 was chosen as the best candidate for testing its applicability for immunohistochemistry.

**Figure 1:**
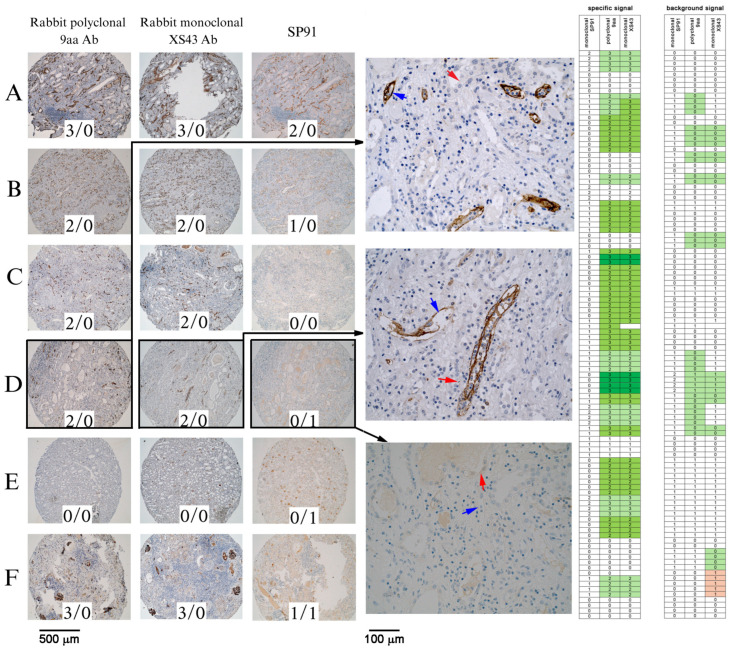
C4d visualization in rejected kidney graft tissues. Left panel: The C4d immunostaining of chosen tissue sections (rows **A**–**F**) with rabbit polyclonal anti-C4d antibody (9aa), monoclonal XS43, and SP91. The values presented in the pictures are the scores for a specific reading vs. background, e.g., 2/0. Middle panel: The slides presented in in row (**D**) of the left panel are shown at a higher magnification (400×) to better illustrate differences between the C4d staining considered specific (a lineage of blood vessels, blue arrowheads) and nonspecific (cytoplasmic staining in epithelial cells of renal tubules, red arrowheads). Right panel: The scoring of the specific C4d signal (left column) and background signal (right column) in 106 tissue microarrays from kidney graft rejections. The readout obtained for the staining with the SP91 clone as used for the routine IVD procedure was considered as a reference. The color code represents an improved (green, in the case of enhancement in a specific signal or lowering the background) or worsened (red, in the case of a background increase) score vs. the SP91 reference.

To properly set the conditions for immunohistochemistry with our polyclonal and monoclonal antibodies, we used the formalin-fixed paraffin-embedded (FFPE) pellets of CD20-positive cells sensitized with complement-activating antibody (rituximab) in conditions permissive (ΔFI + FI) and non-permissive (ΔFI) for C4d formation, as described in [21]. Once the optimal staining conditions were established, we used them consequently to stain tissue microarray (TMA) sections containing explanted kidney graft tissues. The performance of our antibodies (rabbit polyclonal raised against 9aa epitope or rabbit monoclonal XS43) in terms of specific and nonspecific staining was compared to the rabbit monoclonal SP91 antibody certified for in vivo diagnostics and used routinely for the staining of clinical sections. The scoring for both our antibodies was principally the same, but it differed markedly from the scoring for SP91, as presented in the right panel of Figure 1. Not only was their specific signal more intensive than that for SP91 (Figure 1A,B,F), but they also showed a lower background signal (Figure 1D,F). In single cases, C4d deposition was detected in a few cores where SP91 failed, as exemplified in Figure 1C,D. The accuracy of detecting C4d by our antibodies was very clear, and in contrast to the SP91 results, there was no doubt in the interpretation of C4d staining or the assessment of its presence in the studied sections. The complete scoring results for all 106 tissue cores are shown in Figure 1 (right panel).

To find out the reason for the inconsistency between staining with SP91 and XS43 antibodies, we performed a direct ELISA, in which both antibodies were bound to immobilized C4/C4b/C4d proteins. We revealed that SP91 showed a considerably lower signal in wells coated with C4d (Figure S3) compared to the XS43 clone. This could explain the differences in immunohistochemistry scores and the weaker performance of SP91 observed in clinical sections (Figure 1).

## 3. Discussion

We attempted to work on highly specific anti-C4d antibodies a decade ago, as we analyzed the appearance of complement activation markers in patients with B-cell lymphoma and leukemia receiving complement-activating anti-CD20 immunotherapeutics: rituximab or ofatumumab. When using a commercially available ELISA from Quidel to measure C4d in patients’ plasma, the results we obtained were hard to explain logically. For example, there was higher C4d content in the samples collected before drug infusion compared to the samples collected immediately after infusion. Moreover, there was no correlation between C4d content and the markers of the terminal complement pathway, such as C5b-6 or TCC (sC5b-9) [18]. This led us to think that the commercial anti-C4d antibody failed to specifically recognize C4d neoepitopes and motivated us to produce our own anti-C4d antibodies. The two pillars of our strategy were the following: (i) to use a short, five-amino-acid sequence that should constitute a linear neoepitope after the excision of the C4d fragment from iC4b, as an immunogen, and (ii) to use rabbits as the host organism, since rabbits’ immune system is able to generate a larger natural repertoire of high-affinity antibodies compared to mice and since it provides an improved immune response to small antigens [22].

The decision to use a short peptide as an immunogen stems from the assumption that the narrow specificity of complement proteases [23] enables the formation of unique short epitopes that are characteristic only for activated complement components. Anti-C4d antibodies obtained in this way were applied in an ELISA that tested the C4d content in hematological patients’ samples again. This time, the obtained results were highly correlated with both C5b-6 and TCC. Moreover, unlike the commercial C4d ELISA, our test did not show a false positive signal when plasma EDTA samples passed a series of consecutive freeze–thawing cycles or when heat-inactivated serum was used to deposit complement on human antibodies aggregated on a plate [18]. Since that time, our double-affinity-purified monospecific rabbit polyclonal anti-C4d antibodies (or its monoclonal version commercialized by Svar Life Science AB that was raised using the same strategy) were successively used for analyses of complement activation markers in plasma and body fluids in lung cancer [24], scleroderma [25], osteoarthritis and other rheumatic diseases [26,27,28], immune thrombocytopenia [29], COVID-19 [30], systemic lupus erythematosus [31], and B-cell malignancies [18,32], which brought conclusive results, often of prognostic value.

However, we anticipated that the same problem as noticed for the specific detection of the soluble C4d marker referred to the detection of cell-bound C4d by immunohistochemistry. Indeed, several reports have highlighted a problem with the quality of anti-C4d antibodies used in routine clinical diagnostics. For example, in a report on the significance of C4d staining in ABO-compatible liver transplantation, the authors stated that the C4d staining of paraffin sections has limited sensitivity compared to frozen sections and that only a strong and extensive C4d pattern has clinical impact [33]. Nonetheless, a consensus within the C4d staining pattern in liver allograft humoral rejection has still not been made, and possibly, a more accurate antibody devoid of nonspecific binding can overcome these obstacles. The possible cross-reactivity of anti-C4d antibodies to native C4 is of special importance for assessing liver tissue as hepatocytes naturally provide a systemic C4 supply [34]. Therefore, distinguishing between endogenous C4 and exogenous C4d is crucial for proper diagnosis. In the case of kidney allograft rejection, C4d positivity in grafts with significant dysfunction seems to indicate the need for aggressive treatment. Conversely, the clinical significance of C4d presence in grafts with mild or negligible dysfunction is unclear [35]. Importantly, the authors used an anti-C4d antibody from Quidel to stain frozen sections, which was found by us to be nonspecific in certain circumstances and which could potentially contribute to the vague interpretation of C4d positivity in some biopsies. Staying in the field of kidney allografts, Seemayer et al. compared the staining of C4d FFPE sections with frozen ones and concluded that FFPE was characterized by a much lower prevalence and degree of C4d expression than frozen tissue staining [36]. They pointed out that major problems with this method being less accurate are both difficulties in staining interpretation and reduced sensitivity. Other authors also raised the issue of the lack of C4d specificity and were more radical in their conclusions, suggesting its exclusion from being a specific marker in antibody-mediated rejection [37]. For this reason, we suggest the introduction of an upgraded anti-C4d antibody able to overcome the sensitivity issue.

To the best of our knowledge, no study directly compares the performance of different antibodies used for the immunohistochemical staining of C4d. This fact precludes firm conclusions on the suitability of particular antibodies for diagnosing a certain disease or the antibody-mediated rejection of certain transplants. Additionally, C4d staining with these antibodies is rarely optimized for antibody dilution, incubation time, etc., thus providing additional risk for unspecific results. We postulate that every C4d staining method should be validated using cell pellets previously exposed to complement-activating antibodies in C4d-permissive and -non-permissive conditions in a way we described previously [21] and herein. Other researchers, presumably aware of the existence of several anti-C4d antibodies certified for diagnostic use, whose specificity was put in doubt, made an attempt to raise a highly selective anti-C4d antibody [8]. Using a strategy of the structural analysis of epitope and bioinformatic tools, they came to the similar conclusion as we did: (i) that 4–7aa long peptides are the best candidates, and (ii) rabbit is an optimal host. Unfortunately, the exact sequence of their epitope is proprietary and was not revealed.

One of the limitations of our study is the lack of matched data for donor-specific antibodies (DSAs) in the graft recipients’ sera, as they were not available for our cohort. DSAs represent a subset of anti-HLA antibodies considered an established biomarker that predicts antibody-mediated graft rejection [38]. The pathomechanism assumes complement fixation by DSAs and subsequent complement-dependent cytotoxicity attributable to osmotic lysis provoked by the assembly of a membrane attack complex. Numerous observations confirmed a strong correlation between DSA presence and C4d deposition in renal peritubular capillaries [39], even when C4d deposition is minimal [40]. Such cross-validation would help to verify whether the staining with our C4d antibody truly mirrors the acknowledged trigger of antibody-mediated rejection and how it performs compared to the SP91 clone routinely used in diagnostics. Possibly, the enhanced detection of C4d in rejected kidney graft biopsies by XS43 may better correlate with DSA presence, but this must be confirmed in a separate study.

## 4. Materials and Methods

### 4.1. Development of Polyclonal Anti-C4d Antibodies

To search for an optimal neoepitope for monoclonal antibodies, we first performed a screening of custom rabbit anti-C4d polyclonal antibodies for their specificity to the C4d fragment. This part of the project was performed in the framework of external service by Agrisera AB (Vännäs, Sweden). New Zealand White rabbits ≥6 m.o. housed in standard pathogen-permissive conditions were used. Briefly, synthetic peptides corresponding to five (SSTGR), seven (TLSSTGR), nine (NVTLSSTGR), eleven (GLNVTLSSTGR), and thirteen (ERGLNVTLSSTGR) C-terminal amino acids of the C4d fragment were coupled using a double-glycine (GG) linker to keyhole limpet hemocyanine (KLH) and used for rabbits’ immunization. Two rabbits per construct were injected with 200 μg of immunogen in complete Freund’s adjuvant followed by a booster injection with another 200 μg in incomplete Freund’s adjuvant at week 4 and two injections with 100 μg in incomplete Freund’s adjuvant at weeks 8 and 12. Rabbits were bled at week 16. Anti-C4d antibodies were purified by two-step affinity chromatography. In the first step, N-biotinylated variants of peptides used for immunization (produced by Lipopharm, Gdańsk, Poland) were coupled with s HiTrap Streptavidin Sepharose High-Performance column (Cytiva, Washington, DC, USA, cat. no. #17511201), and the fraction eluted with 0.1 M glycine pH 2.5 was collected and neutralized with 1 M Tris-HCl pH 8.0. The eluate was then loaded onto another column coupled to N-biotinylated peptides extended by four amino acids from the C-terminal C4d/C4d boundary (NGFK). The flowthrough from this column was concentrated to the final volume of 1 mL and contained antibodies bondable to the C4d but not the C4b fragment. Such preparations of antibodies were then tested for specificity to C4d in two independent setups of the ELISA.

### 4.2. ELISAs 

Polyclonal antibodies were checked for C4, C4b, and C4d binding in direct ELISA. MaxiSorp plates (96-well, Nunc, Roskilde, Denmark, cat. no. #442404) were coated with 10 μg/mL of C4, C4b (Complement Technology, Tyler, TX, USA, cat. no. #A105 and #A108, respectively), and C4d (produced in-house, as described in [31]) in carbonate buffer and incubated for 1 h at 37 °C. Plates were washed with washing buffer (140 mM NaCl, 20 mM Tris–HCl pH 7.4, 0.2% Tween 20) and blocked for 30 min at 37 °C with 3% fish skin gelatin (Sigma, St. Louis, MO, USA, cat. no. # 404R-1) in washing buffer. After that, they were overlayed with antisera (1:200 in PBS-T buffer) or purified polyclonal rabbit antibodies (1:1000) or rabbit monoclonal anti-C4d antibodies, SP91 (Sigma) and XS43 at concentration 0.1 μg/mL, or clones XS41, SX4 at concentration 25 μg/mL (as at 0.1 μg/mL there was no signal) and incubated for 1 h at 37 °C. Plates were then incubated for 30 min at 37 °C with secondary goat anti-rabbit antibodies conjugated with HRP (Dako, Glostrup, Denmark cat. no. #P0448) in concentration 1:500 in PBS-T buffer for detection. Following washing, tests were developed with the use of 3,3′, 5,5′-tetramethylbenzidine (Sigma) substrate. The reaction was stopped with 0.5 M H_2_SO_4_, and the absorbance at 450 nm was read with a Synergy H1 microplate reader (BioTek, Winooski, VT, USA).

Another ELISA aimed to confirm the specificity of antibodies for C4d deposited in the process of complement activation in human serum. Plates were coated with 20 μg/mL of pentaglobin (a mix of human IgG) for 1 h at 37 °C. After blocking, plates were overlayed with 1% normal human serum (NHS) or factor I-depleted serum (ΔFI; Complement Technology, cat. no. #A338) dilutions in gelatin veronal buffered saline with 0.15 mM CaCl_2_ and 0.5 mM MgCl_2_ (GVB++) or PBS + Ca^2+^ + Mg^2+^ 1:1 with H_2_O. Optionally, ΔFI serum was supplemented with purified factor I (ΔFI + FI; Complement Technology, cat. no. #A138) to reconstitute the physiological content of this protein or anti-FI antibody (Quidel, San Diego, CA, USA, cat. no. #A247) able to block its function. The detection of C4d was carried out in the same manner as described earlier, but XS41 and SX4 concentrations were lowered to 10 μg/mL.

### 4.3. Production of Rabbit Monoclonal Antibodies

After evaluating the optimal C4d neoepitope length, peptides covering the 7 and 9aa long C-terminal residues of C4d were chosen for immunization. The antibody development and production process was delegated to a specialized company (Applied StemCell, Milpitas, CA, USA), which uses single B-cell cloning in Hu rabbits, a genetically modified strain that produces antibodies devoid of additional disulfide in variable parts that are characteristic for rabbit immunoglobulins, thus making them more human-like. We received 40 monoclonal antibody clones, and after the ELISA tests, we decided to proceed with the 3 best-performing clones (XS41, XS43, and SX4). Having plasmids encoding light and heavy chains of chosen antibody clones, we transformed *E. coli* competent cells and purified DNA. Thirty micrograms of purified DNA was used for the transfection of ExpiCHO cells using an ExpiCHO Expression System Kit (Gibco, Paisley, UK, cat. no. #A29133), and purification was performed similarly to polyclonal antibodies but on 1 mL HiTrap Protein A and G columns (Cytiva, cat. no. #17040301 and # 17040501) connected in tandem. Columns were washed with PBS, and antibodies were eluted with 0.1 M glycine buffer, pH 2.5.

### 4.4. Cell Culture

Human lymphoma cell line Raji (ATCC) was cultured in RPMI 1640 medium with L-glutamine (ATCC, Manassas, VA, USA) supplemented with 10% fetal bovine serum (ATCC). Cells were kept in continuous culture at 37 °C in 5% CO_2_ atmosphere for no longer than 10 passages.

The ExpiCHO cell line (Gibco) was cultured in a dedicated ExpiCHO medium at 37 °C in 8% CO_2_ atmosphere with shaking (120 rpm), according to user guidelines. During the transfection and overproduction phases, the temperature was lowered to 32 °C and CO_2_ to 5%.

### 4.5. Positive and Negative Control Cells

To evaluate polyclonal and monoclonal rabbit anti-C4d antibodies as a tool for immunohistochemistry, 10^6^ Raji cells per sample were washed with PBS with 1 mM Mg^2+^/Ca^2+^ and resuspended in 10% factor I-depleted serum (Complement Technology) with the addition of 50 μg/mL of rituximab. These conditions allow for C4b deposition and at the same time, by the lack of factor I, disable C4d formation. Another sample was reconstituted with human recombinant factor I (Complement Technology) at physiological concentration 35 μg/mL to restore the capacity of C4d formation. Cells were incubated for 30 min at 37 °C, with shaking at 650 rpm. After washing with PBS, they were fixed with 4% paraformaldehyde (POCH, Gliwice, Poland) for 4 h at RT and stained with hematoxylin (Sigma Aldrich, St. Louis, MO, USA) for 10 min. Then, they were dehydrated overnight at 4 °C in 70% ethanol, followed by 1 h at RT in 96% ethanol, another 1 h in absolute ethanol, and finally 1 h RT in xylene. The supernatant was discarded, and cell pellets were overlayed with hot paraffin and left in open tubes inside of a thermoblock until all xylene evaporated. Then, the pellets were embedded in paraffin blocks.

### 4.6. Tissue Microarrays

Tissue microarrays (TMAs) were constructed using kidney tissue blocks from 31 patients who had undergone kidney graft explantation, due to rejection, at the University Clinical Center in Gdańsk between 2012 and 2021. This study was approved by the Local Bioethical Committee at the Medical University of Gdańsk (approval number KB/320/2024). TMA samples consisting of 4 representative core sections (1.5 mm in diameter) were prepared using the Manual Tissue Arrayer MTA-1 (Beecher Instruments, Inc., USA). Normal tissues served as a negative control and location markers.

### 4.7. Immunostaining

Paraffin blocks containing cell pellets and microarrays were sectioned using a microtome into 4 μm thick sections and stained using the anti-C4d antibodies. The in vitro diagnostics-validated antibody (SP91, Roche Ventana, Tucson, AZ, USA) was used in automated staining (Ventana Benchmark Ultra, Roche, Tucson, AZ, USA) according to the manufacturer’s protocol (64 min at 95 °C in CC1 buffer and 32 min antibody incubation). The polyclonal anti-C4d antibodies raised against the 9aa long C-terminal epitope (3.5 μg/mL, overnight incubation) and the XS43 rabbit monoclonal antibody (4.2 μg/mL, 60 min incubation) were used in manual staining. For both antibodies, heat-induced epitope retrieval was performed using a pressure cooker and the Target Retrieval Solution pH 9.0 (Agilent, Santa Clara, CA, USA, cat. no. # S236784-2). Scoring was performed by a board-certified pathologist. A sharp staining delineating blood vessels was considered a specific signal, while the staining of the interstitium, tubular cells, or intratubular material was considered a background/nonspecific signal. Both the specific and nonspecific staining intensities were assessed using the routine 4-tiered semiquantitative system (no/weak/moderate/strong signal).

## 5. Conclusions

We showed that either the monospecific polyclonal antibody raised against nine C-terminal amino acids of the C4d fragment or the corresponding rabbit monoclonal antibody generated less background and a clearer staining pattern in its expected locations within the biopsies of antibody-mediated kidney graft rejection, compared to the other anti-C4d antibody approved for routine clinical diagnostics. This result is in line with the results from ELISAs that ensure the on-target binding of the XS43 antibody as well as its polyclonal precursors. Our data provide ground for the examination of different anti-C4d antibodies used for immunohistochemistry in the same set of clinical materials from various pathogenic conditions. Such tests can potentially help to reach more consensus regarding the clinical significance of C4d deposition.

## Data Availability

The raw data supporting the conclusions of this article will be made available by the authors upon request.

## Supplementary Materials

The following supporting information can be downloaded at https://www.mdpi.com/article/10.3390/ijms251910526/s1.
