# Supplementary material for: Accurate Visualization of C4d Complement Fragment in Immunohistochemistry by C-Terminal Linear Neoepitope-Specific Antibodies
Source: Int J Mol Sci. 2024 Sep 30;25(19):10526. doi: 10.3390/ijms251910526 (PMC11476897; doi:10.3390/ijms251910526)
Supplement: Supplementary file 1 [file ijms-25-10526-s001.zip › ijms-3177179-supplementary.pdf]

# Supplementary data

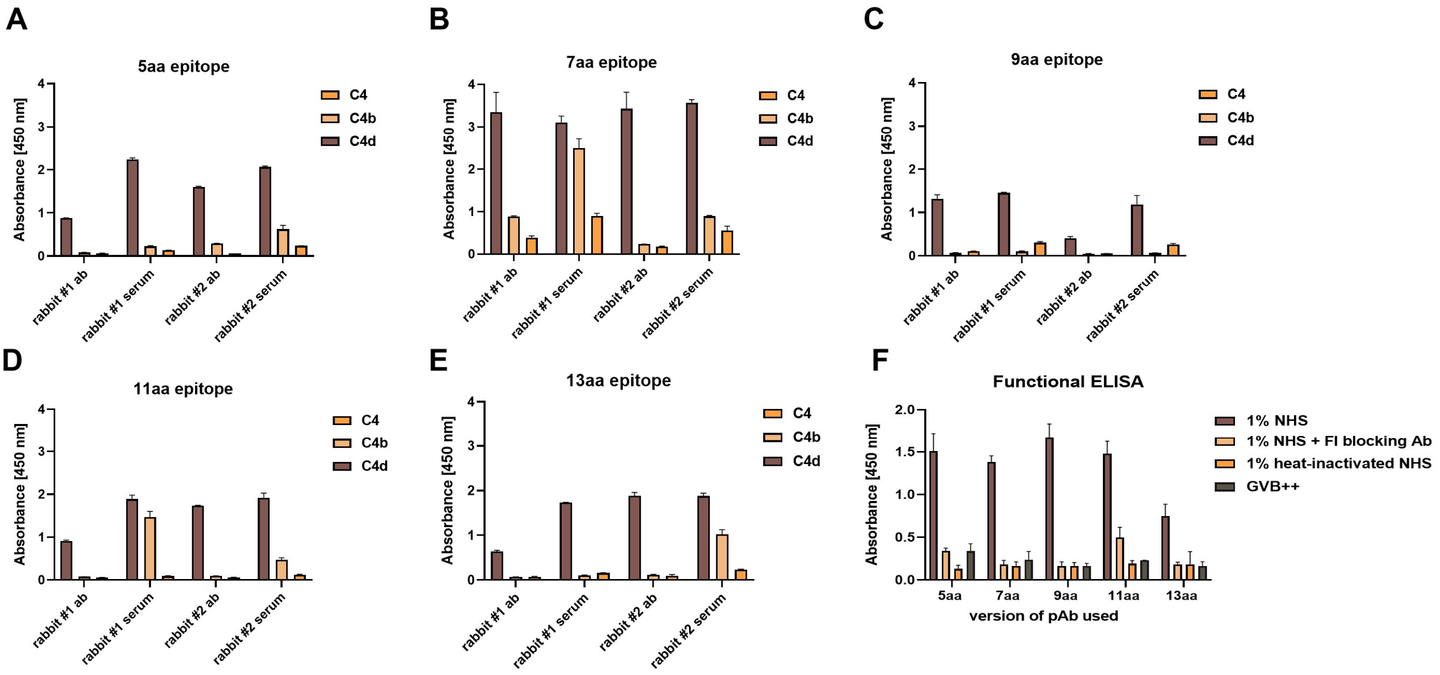


Fig. S1 Assessment of selective C4d binding by polyclonal rabbit antibodies.

In panels A-E plates were coated with C4, C4b, and C4d (10µg/ml) and overlayed with rabbit antisera (1:200) or purified anti-C4d antibodies (1:1000) in order to gauge their specificity and cross-reactivity. Additionally, ability to bind C4d formed in 1% NHS upon classical pathway activation by 20µg/ml of pentaglobin was evaluated (F). FI-blocking antibody was used to prevent C4d generation and thus assess cross-reactivity in more natural-like conditions. Goat anti-rabbit antibodies conjugated with HRP were used for detection. Test were developed and absorbance at 450 nm was read. Standard deviation presented by error bars was calculated from two technical repeats.

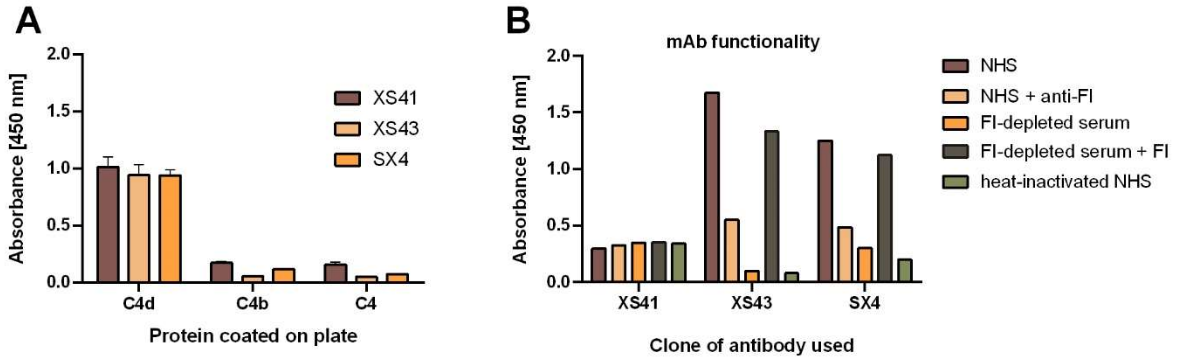


Fig. S2 C4d detection by monoclonal rabbit antibodies.

A. C4, C4b and C4d (10µg/ml) were coated on a plate and overlayed with anti-C4d antibodies XS41, SX4 (25 µg/ml) and XS43 (0.1 µg/ml) in order to assess their specificity and check for cross-reactivity. To evaluate their ability to bind C4d formed during classical pathway activation, plates were coated with 20µg/ml of pentaglobin followed by 1% NHS incubation (B). FI-blocking antibody was used to prevent C4d generation and thus assess cross-reactivity in more natural-like conditions. Additionally, two more conditions were tested; one in which C4d cannot be formed (1% ΔFI serum) and second where FI is reconstituted to physiological concentration (ΔFI + FI). Goat anti-rabbit antibodies conjugated with HRP were used for detection. Test were developed and absorbance at 450 nm was read. Standard deviation presented by error bars was calculated from duplicates in panel A, ELISA in panel B was performed with single measurements.

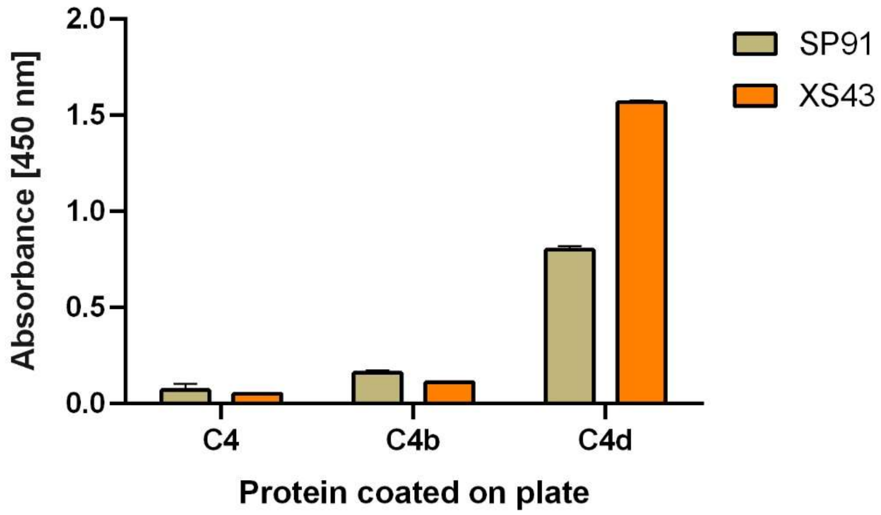


Fig. S3 ELISA assay evaluating commercial anti-C4d antibody SP91 specificity versus XS43 clone.

Plates were coated with purified C4, C4b and C4d at concentrations 10 µg/ml and overlaid with SP91 and XS43 at 0.1 µg/ml concentrations. Goat anti-rabbit antibodies conjugated with HRP were used for detection. Tests were developed and absorbance at 450 nm was read. Standard deviation presented by error bars was calculated from duplicates.
